# Effect of Ultrasound Combined with Plasma-Activated Water on Lethal and Sublethal Injury Against *Escherichia coli*

**DOI:** 10.3390/foods14091457

**Published:** 2025-04-23

**Authors:** Xin Wen, Meimei Nie, Zhongyuan Zhang, Lingming Xiong, Jialin Feng, Zhi Zhang, Dajing Li, Yihong Bao, Haihong Wu

**Affiliations:** 1 College of Life Sciences, Northeast Forestry University, Harbin 150040, China; wx99074@163.com (X.W.); linaishenqi@163.com (J.F.); zz2522490443@163.com (Z.Z.); 2 Institute of Agro-Product Processing, Jiangsu Academy of Agricultural Sciences, Nanjing 210014, China; nmm1011@163.com (M.N.); zzyszy2012@163.com (Z.Z.); xionglinming219@163.com (L.X.); lidajing@163.com (D.L.)

**Keywords:** plasma-activated water, ultrasound, *E. coli*, antimicrobial mechanism, sublethal injury, oxidative stress

## Abstract

Plasma-activated water (PAW) treatment is a promising technique for food processing, but it causes sublethal injury (SI) to microorganisms. This study investigated the effect of ultrasound (US) combined with PAW (US-PAW) on SI of *Escherichia coli* (*E. coli*). Results showed that, after plasma activation for 10 min and treatment for 10 min, the US-PAW treatment caused a 4.89 ± 0.07 log CFU/mL reduction in *E. coli*. Meanwhile, under these conditions, the SI rate of *E. coli* was decreased to 13.3 ± 2.15%, significantly reduced by 52.74% compared to using PAW alone. The inactivation process of US-PAW treatment fitted the Weibull model better. The morphology of *E. coli* was destroyed by PAW and US-PAW treatment. Additionally, US-PAW treatment significantly increased the leakage of protein and nucleic acid, as well as cell membrane permeability and potential. Compared to PAW or US treatment, the proportion of membrane fatty acids and the structure of membrane proteins were altered in the US-PAW group. Furthermore, intracellular reactive oxygen species (ROS) levels increased by US-PAW treatment, and the levels of GSH, SOD, and CAT enzyme activities were significantly reduced, compared to PAW or US treatment. The combined treatment also resulted in significant DNA oxidative damage. The disruption of cell membrane structure and oxidative damage caused by US-PAW treatment resulted in irreversible damage to bacteria, thus reducing the SI rate.

## 1. Introduction

Foodborne pathogens are microorganisms that can be transmitted through food and have the potential to cause severe illnesses, including food poisoning, toxic infections, and other related diseases, posing a significant public health risk [1]. The most effective strategy of preventing foodborne illness is to control food contamination and improve the technical means of food sterilization. *Escherichia coli* (*E. coli*) is a common foodborne pathogen, belonging to the family *Enterobacteriaceae* [2]. According to the World Health Organization (WHO) [3], approximately, there are nearly 1.7 billion cases of childhood infection with diarrheal disease owing to *E. coli*, and diarrhea kills around 443,832 children under 5 years. At present, non-thermal sterilization technology is gaining increasing popularity, because it can be efficient as a sterilization method, but also can maintain the nutritional and sensory quality of food. These technologies include the high-voltage electrostatic field, pulsed electric field, ultrasound (US), and plasma-activated water (PAW) [4]. PAW is a prospective alternative disinfectant, prepared by non-thermal plasma system discharged in water, containing various antibacterial compounds [5]. PAW has the characteristics of high efficiency and a broad spectrum, safety and no residue, and simple operation, and is suitable for use in food processing. Reactive oxygen species (ROS) and reactive nitrogen species (RNS) play a major role in PAW processing [6]. However, a single PAW treatment cannot completely inactivate microorganisms. Pan et al. investigated the effect Ar/O_2_ plasma treatment on *Listeria monocytogenes* (*L. monocytogenes*); results displayed that the proportion of sublethal injury (SI) cells exhibited time-dependent behavior [7].

SI refers to a state of damage in microorganisms that falls between survival and death [8]. SI cells may repair themselves and regrow under favorable conditions, thus causing potential food safety risks [9]. Shao et al. [10] revealed that the injured *Staphylococcus aureus* (*S. aureus*) cells by ohmic heating were fully repaired in nutrient broth at 37 °C and pH 7.2 and the expression levels of virulence genes increased during this process. At present, the microorganisms that can form SI, such as *E. coli*, *S. aureus*, and *L. monocytogenes* [11]. Numerous studies have shown that high-pressure carbon dioxide, high-pressure processing, and others can induce SI in microorganisms [12,13,14]. When *E. coli* cells were exposed to slightly acidic electrolyzed water (SAEW), the sublethal ratios increased with treatment volume [15]. The latest research has shown that synergistic treatment with different non-thermal processing techniques can significantly reduce the number of SI cells.

The combination of US and PAW (US-PAW) is a promising hurdle technology with enormous potential in food processing. Royintarat et al. found that US-PAW treatment was more effective curbing *E. coli* and *S. aureus* on chicken meat and skin than the use of only one approach [16]. In the treatment of *E. coli* inoculated on grass carp, Johnson Esua et al. reported that US combined with PAW showed an approximately 0.6 log CFU/g reduction compared to PAW alone [17]. Recent studies have shown that compared to a single treatment, US-PAW processing significantly reduced the surface microbial community of crayfish and maintained the quality of the storage period [18]. From these reports [16,17,18], the bactericidal efficacy of PAW was improved by being combined with US treatment. However, the effect of PAW combined with US on controlling the SI rate of microorganisms was not clear. Several reports have proved that US treatment alone does not cause sublethal injury, but efficiency of microbial inactivation is low [19,20,21]. It is worthwhile to explore the improvement in inactivation rate while controlling the SI rate through the combined treatment of US and PAW.

Therefore, the objective of the present work was to investigate the effect of US-PAW on log reduction and the SI rate of *E. coli*, as well as the possible mechanism. From the aspect of cell membrane structure, effect on the cell membrane integrity, permeability, membrane potential, the membrane fatty acid and protein structure were observed. On the other hand, the content of intracellular ROS, the antioxidant active substances, and DNA oxidant level were explored. The research results are of great significance for improving the inactivation performance and controlling the sublethal damage of non-thermal processing combined treatment in practical applications.

## 2. Materials and Methods

### 2.1. Bacterial Strains Culture

*E. coli* strain (ATCC25922) was inoculated into trypsin soybean agar (TSA) (Hopebio Technology Co., Ltd., Qingdao, China), where it was cultivated for 24 h, and individual colonies were selected and inoculated in sterile trypsin soybean broth (TSB) (Hopebio Technology Co., Ltd., Qingdao, China). The bacterial cells were harvested by centrifugation at 5000× *g* for 5 min and resuspended in sterile saline. The final bacterial density was about 10^8^ CFU/mL.

### 2.2. Preparation of PAW

A non-thermal atmospheric pressure plasma system (PG-1000Z/D, Nanjing Suman Electronics Co., Ltd., Nanjing, China) was used to generate PAW. The system consisted of plasma jet nozzle, a high-voltage generator (800 W), and gas control device. Using compressed air at 0.18 MPa as working gas with a flow rate of 20–30 L/min, the plasma jet probe was penetrated into the liquid surface below 5 mm. Sterile distilled water, with each sample being 300 mL, was used for activation under cold plasma for 5, 10, 15 min, defined as PAW5, PAW10, PAW15, respectively.

### 2.3. Samples Treatment

The US treatment was performed on a 4 L US tank (KQ-100DE, Ultrasonic Instrument Co., Ltd., Kunshan, China). In the experiment, the frequency was set to 40 kHz, the power was 250 W, the temperature was maintained below 25 °C.

**Figure 1:**
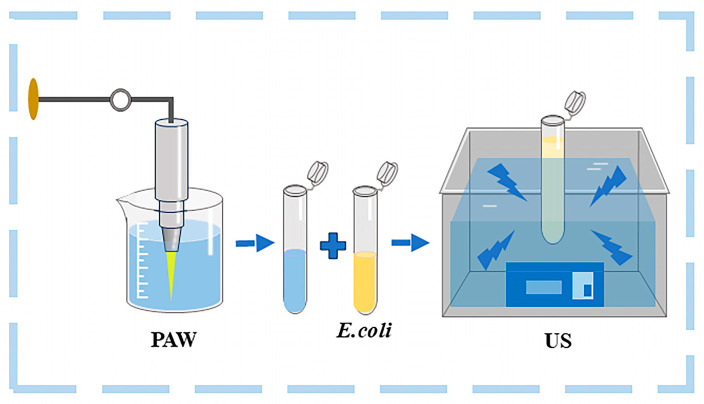
A schematic diagram of US combined with PAW processing against *E. coli*.

For the PAW treatment, 4 mL of bacterial suspension and an equal volume PAW were mixed. The control group was treated with sterile saline instead of PAW. The bacterial suspension being mixed with or without PAW was placed in the centrifuge tube in the US tank for US-PAW or US treatment (see Figure 1). After being treated for 3, 5, 7, or 10 min for all groups, the bacterial suspension was then put through the subsequent test.

### 2.4. Inhibition Efficiency

The bacterial suspension was a tenfold gradient diluted with 0.85% normal saline, inoculated on non-selective medium (TSA) and selective medium (TSA with 3% NaCl, Hopebio Technology Co., Ltd., Qingdao, China), respectively. The entire plate was incubated at 37 °C for 48 h and counted. The lethal and sublethal effects of the synergistic treatment on *E. coli* were obtained by log reduction and SI rate. The synergistic bactericidal effect was compared by synergistic inhibition rate [22]. The formulae are as follows:

log reduction = log_10_(N/N_0_), (1)

where N represents the number of colonies on TSA medium before treatment and N_0_ represents the number of colonies after treatment in CFU/mL.

SI rate = (A − A_0_)/A × 100%, (2)

where A represents the number of colonies on non-selective media for different treatments and A_0_ represents the colonies on selective media for different treatments in CFU/mL.

Synergistic inhibition = ΔI = I_AC_ − (I_A_ + I_C_), (3)

where I_A_, I_C_, and I_A+C_ are the log reduction after PAW, US, and US-PAW processing, respectively.

### 2.5. Inactivation Kinetics Model

#### 2.5.1. Linear Model

This model supposes a linear relationship between the number of residual microorganisms and processing time, as shown in the following formula:

log_10_(N/N_0_) = −t/D (4)

N and N_0_ are the number of colonies (CFU/mL) before and after different treatments, respectively. D is the time need to kill 90% of microorganisms in min. t is the processing time in min.

#### 2.5.2. Weibull Model

The model was progressed by Peleg and Cole [23] as the following formula:

Log_10_(N/N_0_) = −(t/σ)^ρ^ (5)

N and N_0_ are the number of colonies (CFU/mL) before and after different treatments, respectively. σ is a scale parameter. ρ is a shape parameter: when ρ < 1, the curve is concave upwards, when ρ > 1, the curve is concave downward, and when ρ = 1, the curve is a line.

### 2.6. Scanning Electron Microscopy (SEM)

The treated bacterial suspension was centrifuged at 4 °C, 12,000× *g* for 2 min; the cells were fixed in 2.5% glutaraldehyde at 4 °C for 12 h [24], followed by washing with 0.1 M PBS. The cells were eluted by a gradient of ethanol solution for 10 min each. Afterwards, the cells were washed with isoamyl acetate for 15 min each time, instead of ethanol. The prepared samples were sprayed with gold and observed via SEM (EVO-LS10, ZEISS Co., Ltd., Jena, Germany) at 10 kV.

### 2.7. Cell Membrane Structure Observation

#### 2.7.1. Determination of Cell Membrane Integrity

The cell was collected by centrifugation (8000× *g*, 10 min). The leakage of nucleic acids and proteins from the supernatants was analyzed by measuring the absorbance at 260 nm and 280 nm, respectively, with a UV–VIS spectrophotometer (UV-6300, Mapada Instruments Co., Ltd., Shanghai, China) [25].

#### 2.7.2. Determination of Cell Membrane Permeability

After centrifugation, the cell was incubated with PI (Solarbio Science&Technology Co., Ltd., Beijing, China) at a final concentration of 3 μM at room temperature in the dark. The fluorescence intensity was determined at excitation and emission wavelengths of 535 nm and 615 nm (Spark, Tecan Trading Co., Ltd., Männedorf, Switzerland), respectively. The cell membrane permeability of *E. coli* cells was expressed as relative fluorescence intensity (RFI) [26].

RFI = F_1_/F_0_ × 100%, (6)

F_0_ and F_1_ represent the PI fluorescence intensity of control and treated group, respectively.

#### 2.7.3. Determination of Cell Membrane Potential

Cells were incubated with DiBAC_4_(3) (Macklin Biochemical Technology Co., Ltd., Shanghai, China) at a final concentration of 2.5 µg/mL at room temperature for 30 min in the dark. Centrifugation was performed at 4 °C, 12,000× *g* for 2 min. Cells were washed and resuspended in PBS. The fluorescence intensity was measured by fluorescence spectrophotometer (Cary Eclipse, Agilent Technologies Co., Ltd., Santa Clara, CA, USA). The excitation and emission wavelength was set at 485 nm and 525 nm, respectively [27].

#### 2.7.4. Analysis of Membrane Fatty Acid Composition

According to a previous study [28], after centrifugation, the cell precipitate was washed three times with saline. Chloroform–methanol (Tedia Company Inc., Fairfield, OH, USA) solution (chloroform/methanol = 1:2, *v*/*v*) was added and shaken for 15 min. Subsequently, chloroform and deionized water were added and shaken. The samples were centrifugated at 6000× *g* for 10 min, the lower phase removed, and dried with a nitrogen blower for 30 min. Next, sodium methylate (Macklin Biochemical Technology Co., Ltd., Shanghai, China) solution was added, followed by shaking for 5 min with ice to prepare the methyl ester. Finally, the methyl ester of the extracted fatty acid was mixed with hexane and shaken. The sample was centrifuged at 6000× *g* for 5 min, and the upper clear phase transferred into a gas chromatography (GC) vial using a 0.22 μm filtration membrane for analysis.

#### 2.7.5. Membrane Protein Structure

*E. coli* membrane protein structure was determined using a previously reported method [29]. The treated cells were washed and resuspended in 0.85% saline solution with a cell density of approximately 10^8^ CFU/mL. Subsequently, potassium iodide (KI, Sinopharm Chemical Reagent Co., Ltd., Shanghai, China) solutions of diverse concentrations were utilized as the positive control and kept at 25 °C for 1 h. The emission spectra were obtained by scanning from 280 nm to 400 nm with a fluorescence spectrophotometer (Cary Eclipse, Agilent Technologies Co., Ltd., Santa Clara, CA, USA). The excitation wavelength was fixed at 258 nm, 280 nm, and 296 nm.

### 2.8. Measurement of Oxidative Damage Indexes of E. coli

#### 2.8.1. Intracellular ROS Content

DCFH-DA (Solarbio Science & Technology Co., Ltd., Beijing, China), a cell-permeable probe, was used to estimate intracellular ROS content of *E. coli* cells. A concentration of 5 µM DCFH-DA was added to the bacterial suspension and maintained for 30 min at room temperature in the darkness. Thereafter, *E. coli* cells were centrifuged (12,000× *g*, 2 min), washed and then redissolved in PBS [30]. The fluorescence intensity was measured using a fluorescence spectrophotometer (Cary Eclipse, Agilent Technologies Co., Ltd., Santa Clara, CA, USA). The excitation and emission wavelengths were set to 488 nm and 525 nm, respectively.

#### 2.8.2. Determination of Oxidative Damage in *E. coli*

Centrifuged at 3500× *g* for 10 min, the treated bacteria were washed and redissolved in PBS. *E. coli* cells were broken by sonication and centrifuged. The levels of intracellular glutathione (GSH), superoxide dismutase (SOD), and catalase (CAT) in treated *E. coli* cells were determined using relevant detection kits (Jiancheng Bioengineering Institute Co., Ltd., Nanjing, China).

#### 2.8.3. Determination of DNA Oxidative Damage in *E. coli*

After centrifugation (1000× *g*, 10 min), the treated bacteria were washed and redissolved in PBS. *E. coli* cells were broken by sonication and centrifuged. Oxidative DNA damage in treated *E. coli* cells were determined using 8-Hydroxydeoxyguanosine (8-OHdG) ELISA kit (Sangon Biotech Co., Ltd., Shanghai, China).

### 2.9. Statistical Analysis

Data were expressed as mean ± SD of three independent biological replicates (*n* = 3). Analysis of variance (ANOVA) and Duncan’s multiple range test were conducted on SPSS 26.0 software (SPSS Inc., Chicago, IL, USA); significant differences between samples are expressed as *p* < 0.05. Origin Pro 2024 software was used for plotting.

## 3. Results and Discussion

### 3.1. Effect of US-PAW Treatments on Lethal and Sublethal Injury on E. coli

As seen in Table 1, the log reduction in *E. coli* treated with US, PAW, and US-PAW at different processing times and PAW activated times were investigated. No significant (*p* > 0.05) inactivation effect of *E. coli* was observed following individual US treatment. For PAW treatment, when plasma activated time increased from 5 to 15 min, the microbial reduction value showed an increasing trend. Among them, after 10 min of treatment, the log reduction in the PAW15 group was 5.62 times that of PAW5. The PAW prepared by mixing the surface discharge-activated air with helium as the working gas of the jet can kill *E. coli* at more than five orders of magnitude [31]. Pronounced increases were observed when a joint simultaneous application of US and PAW was employed. When plasma-activated for 15 min and treated for 3 to 10 min, the log reduction in *E. coli* obtained by US-PAW processing varied from 1.25 ± 0.14 to 5.24 ± 0.40 log CFU/mL, exceeding that of the PAW group from 1.03 ± 0.15 to 4.83 ± 0.07 log CFU/mL. At 10 min, the log reduction in the combined group of 4.89 log CFU/mL was remarkably higher than that in PAW treatment of 3.59 log CFU/mL when plasma-activated for 10 min. No significant (*p* > 0.05) effect was observed between the PAW group and the US-PAW group after 10 min of plasma activation and 7 min of treatment.

The SI rate was evaluated by selective medium method. As shown in Table 2, the SI rate of *E. coli* treated with US for 0–7 min increased significantly (*p* < 0.05). As the PAW treatment time prolonged, the SI rate of *E. coli* significantly decreased (*p* < 0.05). When PAW15 was used to treat for 10 min, the SI rate decreased by 47.49% compared to 3 min of treatment. At the same time, with the increase in PAW activation time, the SI rate showed an increasing trend. Under the condition of PAW activation for 10 min, no significant (*p* > 0.05) difference in SI rate was shown between PAW treatment alone and US-PAW treatment for 3 min and 5 min. However, US-PAW treatment for 7 min and 10 min significantly (*p* < 0.05) reduced SI rates of *E. coli*. This may be because as the time prolonged, the accumulated damage owing to combined treatment exceeded the stress response ability of the bacteria, resulting in bacterial death. The SI rate of the US-PAW10 group at 10 min remained 13.3 ± 2.15%, which was significantly (*p* < 0.05) lower than that of other groups.

Previous studies have shown that PAW has good bactericidal effects, but the influence on sublethal damage was still not clear. Thus, this experiment explored the inactivation effect of PAW combined with US treatment on *E. coli*, while also paying attention to its impact on SI rate. After US treatment for 7 min, the SI rate reached a higher value. Possibly due to the prolonged US-treated time, the tolerance of *E. coli* to the external environmental conditions increased, and the SI rate increased. According to Li et al., a single US treatment for 10 min induced the formation of SI of *S. aureus* to 45.75% [20]. Although the SI rate of US treatment was relatively high at this condition, overall, it still reduced the SI of combined treatment.

The sterilization mechanism of US was that it generated a cavitation effect, dispersed cells, and made bacteria more susceptible to external pressure, releasing cell contents [32]. The main mechanism of PAW sterilization was to increase cell membrane penetration, allowing for active substances such as ROS to further destroy the cell structure [33]. In the present study, from Table 1, the bacterial log reduction values of in the US treatment group were lower, not exceeding 1 log CFU/mL, while the log reduction values in most US-PAW groups were significantly higher than in the PAW alone group. This confirmed that PAW processing played the main role in killing *E. coli* in the combined treatment, while after plasma activation for 10 and 15 min, compared to the PAW group during 10 min treatment, the SI rate was significantly reduced by 52.74% and 27.62% in the US-PAW group, respectively. It was due to that in collaborative processing, PAW was more likely to damage the cell wall, leading to an increase in SI rate. As the duration of collaborative treatment prolonged, US accelerated the overflow of cellular contents, further accelerating cell death and reducing the rate of SI. This indicated that US processing played a principal role in decreasing the SI rate. Thus, US combined with PAW treatment could achieve a synergistic bactericidal effect, meanwhile effectively controlling the SI rate.

### 3.2. Synergistic Inhibition of Combined Treatment

**Figure 2:**
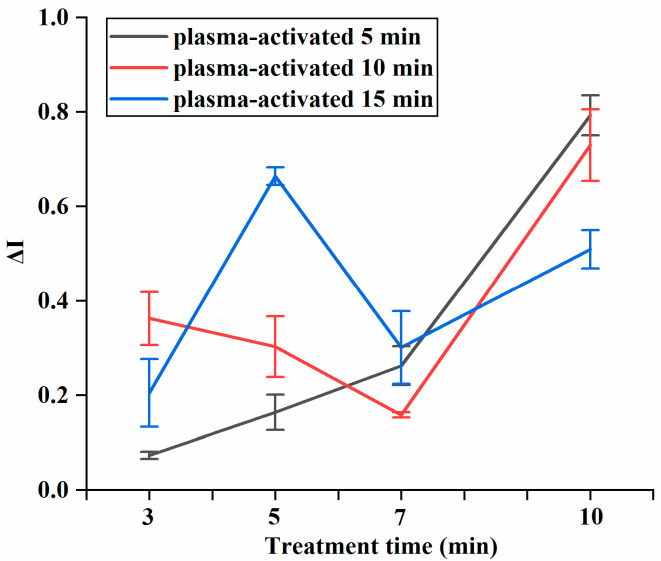
Synergistic inhibition of combined treatment at different plasma activation times.

The final synergistic parameter result was greater than zero, indicating a synergistic effect between the two treatments. A higher value suggests a more pronounced synergistic effect. As shown in Figure 2, the synergistic inhibition values obtained were all greater than 0, demonstrating that US acts synergistically with PAW to effectively target *E. coli*. Specifically, when cold plasma-activated for 15 min, the synergistic inhibition value showed a gradually increasing trends with treatment time. When *E. coli* was plasma-activated for 10 min and treated for 10 min, the synergistic inhibition index reached 0.73 ± 0.08, significantly (*p* < 0.05) higher than other treatments. This suggests that it obtained a better synergistic effect under these conditions. In addition, the synergistic inhibition value reached a turning point at 7 min, and the synergistic inhibition value at different plasma activation times all increased significantly after 7 min, indicating an enhanced synergistic effect. Therefore, plasma activation for 10 min and treatment for 10 min was set as the better condition for the next exploration of mechanism experiments.

### 3.3. Kinetic Model Fit Analysis

**Figure 3:**
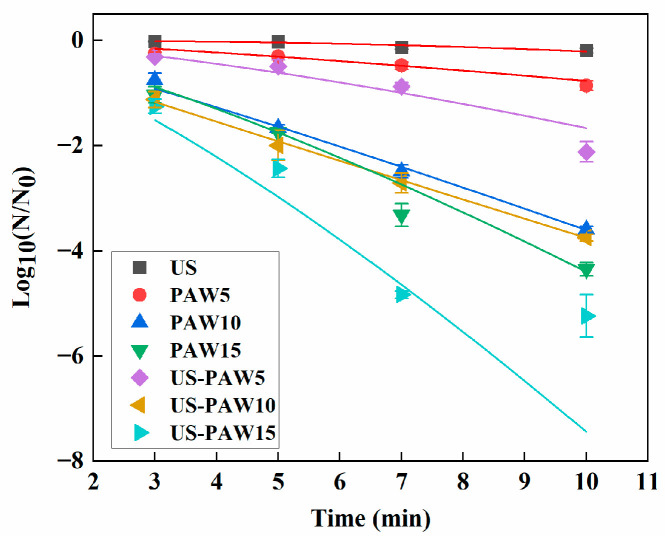
The Weibull model of *E. coli* with different treatment times and different treatment methods. US: ultrasound. PAW: plasma-activated water. US-PAW: US combined with PAW. The numbers 5, 10, and 15 represent plasma activation at 5 min, 10 min, and 15 min.

The linear model and Weibull model were used to evaluate the mechanical model of deactivation of *E. coli* with different treatment methods over time. The fitting results and parameters are shown in Table 3 and Figure 3. The relatively low R^2^ values indicate that the fitting property of the linear model is not optimal. On the contrary, the R^2^ values obtained from the Weibull model, which ranged from 0.8127 to 0.9992, obviously surpassed those of the linear model. The shape factor ρ of the inactivation curves of other treatments were all greater than 1, except for US-PAW5, which indicated that the inactivation curves presented an upward concave degree. Parameter σ was lower in the US-PAW treatment than in the US and the PAW treatment alone, suggesting that the US-PAW treatment needed less time for the inactivation of *E. coli*. This accorded with the basic requirements for hurdle technology. Previously, Weibull models were successfully used to describe survival curves corresponding to microbial inactivation by cold plasma. Pan et al. applied the Weibull model to better assess the plasma-mediated inactivation kinetics of *L. monocytogenes* cells compared to the linear model [7]. In addition, a previous study showed that the D value of *E. coli* was 1.38 min in hot water at 70 °C [34]. The D value of the US-PAW10 group was 2.78 min. Although the absolute D value was high, this hurdle technology could achieve bacterial reduction efficiently at room temperature and avoid damage to heat-sensitive materials. This suggests that the Weibull model demonstrated excellent fitting ability for the inactivation curves of *E. coli* induced by US, PAW, and US-PAW.

### 3.4. SEM Observations

**Figure 4:**
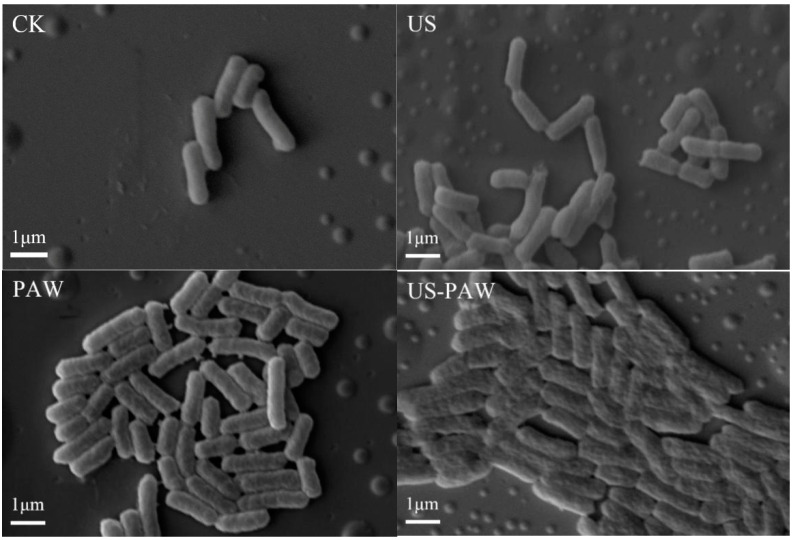
Effect of different treatments on cell morphology in *E. coli*. CK: control group. US: ultrasound. PAW: plasma-activated water. US-PAW: US combined with PAW. The scale bar is 1 µm. Original magnification: ×10,000.

As shown in Figure 4, *E. coli* in the control group exhibited a regular rod shape, with full and uniform cells, and a complete and smooth surface. After US treatment, the morphology of *E. coli* bacteria remained essentially unchanged. After PAW alone treatment, the *E. coli* cells were slightly crumpled, and their surfaces had turned rough, while the surface of *E. coli* cells presented obvious folds and depressions with US-PAW treatment. The aforementioned results indicate that the US-PAW collaborative treatment impaired the cell morphology of *E. coli*, which might further influence its normal physiological metabolism and eventually result in its inactivation. After being treated with PAW and propylparaben, significant morphological changes were also observed in *E. coli* O157:H7 cells [35].

### 3.5. Effect of US-PAW Treatment on Cell Membrane Structure

#### 3.5.1. Determination of Cell Membrane Integrity

**Figure 5:**
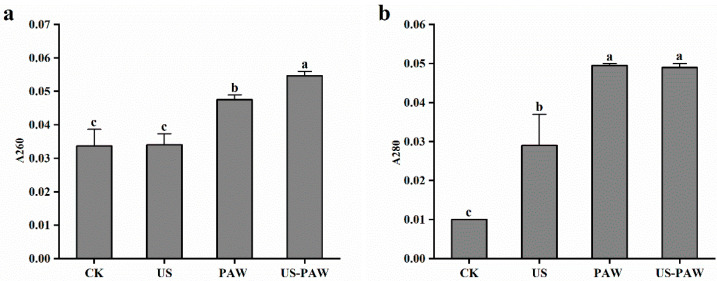
Effect of different treatments on the absorbance values of intracellular nucleic acid (**a**), protein (**b**) in *E. coli*. Values are shown as the means ± SD. Different small letters in each group indicate significant (*p* < 0.05) statistical differences. CK: control group. US: ultrasound. PAW: plasma-activated water. US-PAW: US combined with PAW.

Nucleic acid and protein have characteristic peaks in the UV region at 260 nm and 280 nm, respectively, and their concentrations are proportional to the absorption value. Therefore, the integrity of cell membrane can be inferred from the leakage of extracellular nucleic acid and protein [36]. As shown in Figure 5, there was a small amount of leakage in the control group, which may be caused by the death of certain cells during their normal life cycle. Compared to the control, the amount of *E. coli* nucleic acid leakage treated by US was not significant (*p* > 0.05). After PAW and US-PAW treatment, the absorbance value at 260 nm was significantly (*p* < 0.05) higher than CK, indicating that the PAW treatment and US-PAW treatment promoted nucleic acid leakage. Compared to PAW single treatment, the 260 nm absorbance in US-PAW treatment was significantly (*p* < 0.05) increased, reaching 0.05. The protein leakage changing trend was consistent with nucleic acid.

Presumably, it was because the shockwave generated by the rupture of bubbles formed by cavitation of US created instantaneous pores on the cell membrane [37], thereby making it easier for PAW to penetrate into the cell and accelerating the nucleic acid and proteins to flow out of the cell. Coupled with SEM graphs, a higher degree of cell membrane breakage was induced by the combined treatment. This may be the main reason for the significant increase in leakage volume under synergistic treatment.

#### 3.5.2. Determination of Cell Membrane Permeability

**Figure 6:**
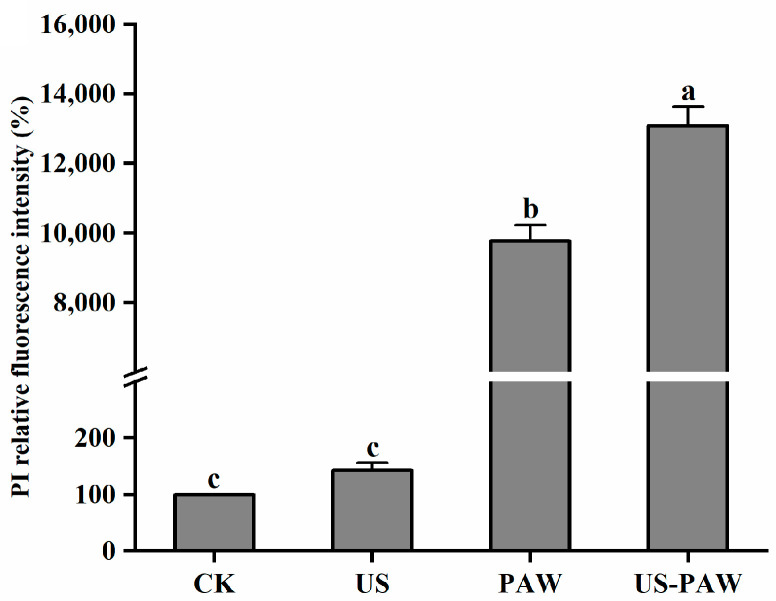
Effect of different treatments on membrane permeability in *E. coli*. Values are shown as the means ± SD. Different small letters in each group indicate significant (*p* < 0.05) statistical differences. CK: control group. US: ultrasound. PAW: plasma-activated water. US-PAW: US combined with PAW.

The permeability of the treated *E. coli* cell membrane was assessed by measuring the relative fluorescence intensity of PI [38]. As shown in Figure 6, US treatment did not significantly change (*p* > 0.05) the relative fluorescence intensity of *E. coli* compared to the control group. The fluorescence intensity significantly (*p* < 0.05) increased after PAW alone and the combined treatment. In addition, the relative fluorescence intensity of PI in US-PAW treatment was 1.34 times higher than that in PAW treatment (*p* < 0.05).

The antibacterial effect of PAW is closely related to its physicochemical properties, including pH value, conductivity, and ORP [32]. The effect of US on the physicochemical properties of PAW under different activated times was measured in the present study. The pH of US-PAW10 group was 2.49 ± 0.02, the conductivity was 1761.33 ± 35.25 μS/cm, and the ORP was 466.90 ± 3.51 mV, all of which were significantly higher than those of PAW10 group, which was consistent with published reports [33], US treatment increased ORP, as well as the content of O_3_, NO^2−^, and OH⋅ in PAW. These highly oxidizing substances to some extent altered the permeability of bacterial cell membranes [39]. This may be the main reason for antibacterial effect under synergistic treatment.

#### 3.5.3. Determination of Cell Membrane Potential

**Figure 7:**
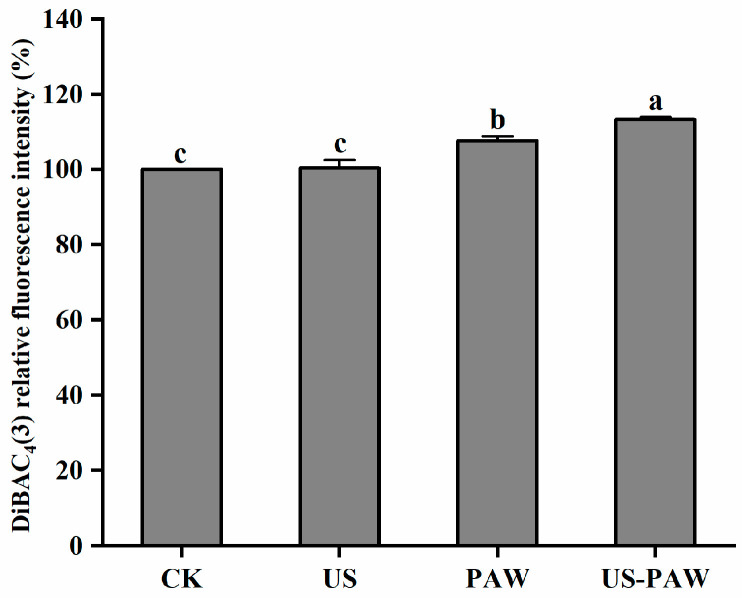
Effect of different treatments on the membrane potential in *E. coli*. Values are shown as the means ± SD. Different small letters in each group indicate significant (*p* < 0.05) statistical differences. CK: control group. US: ultrasound. PAW: plasma-activated water. US-PAW: US combined with PAW.

The membrane potential refers to the potential difference between the two sides of the cell membrane and plays a crucial role in regulating the physiology and behavior of bacteria [40]. As depicted in Figure 7, compared with the CK, the relative fluorescence intensity was no significant different after US treatment. After PAW and US-PAW treatment, the relative fluorescence intensity was increased significantly (*p* > 0.05), by 7.69% and 13.32%, respectively. The result indicated that the cell membrane was in a depolarization state.

Research has shown that depolarization of the cell membrane can affect ion channels such as K^+^, Na^+^, and Ca^2+^ inside the cell, leading to an increase in the potential difference between the inside and outside of the bacterial cell membrane, causing irreversible damage to the cell and ultimately inducing cell death [41,42]. This result was consistent with the tendency in cell membrane permeability and microbial log reduction.

#### 3.5.4. Analysis of Membrane Fatty Acid Composition

The effect of US combined PAW treatment on composition of fatty acids in *E. coli* cell membrane were detected by GC-MS. As Table 4 shows, there were mainly 10 types of fatty acids in the *E. coli* cell membrane. These fatty acids can be broadly divided into two groups: saturated fatty acids (SFAs), including C4:0, C8:0, C12:0, C14:0, C15:0, C16:0, C17:0, C18:0, and C22:0, and unsaturated fatty acids (UFAs), including C16:1, C18:1, and C18:2. Compared to the CK, there was an increase in the proportion of SFAs and a decrease in the proportion of USFAs following US treatment. Compared to CK, the proportion of SFAs in US-PAW treatment was significantly reduced from 79.67 ± 0.50% to 74.24 ± 0.19% and the proportion of UFAs was significantly increased from 20.49 ± 0.33% to 25.76 ± 0.19%. In particular, the reducing of proportion of SFAs was mainly due to butyric acid (C4:0); after US-PAW treatment, the level of butyric acid declined by 57.30%, whereas the increasing proportion of UFAs was mainly attributed to the content of linoleic acid (C18:2), which rose significantly from 3.56 ± 0.44% to 4.46 ± 0.21%. In addition, the proportion of SFAs in the US-PAW group was significantly reduced by 5.15% and the proportion of UFAs was significantly increased by 18.55% compared with the PAW group.

**Figure 8:**
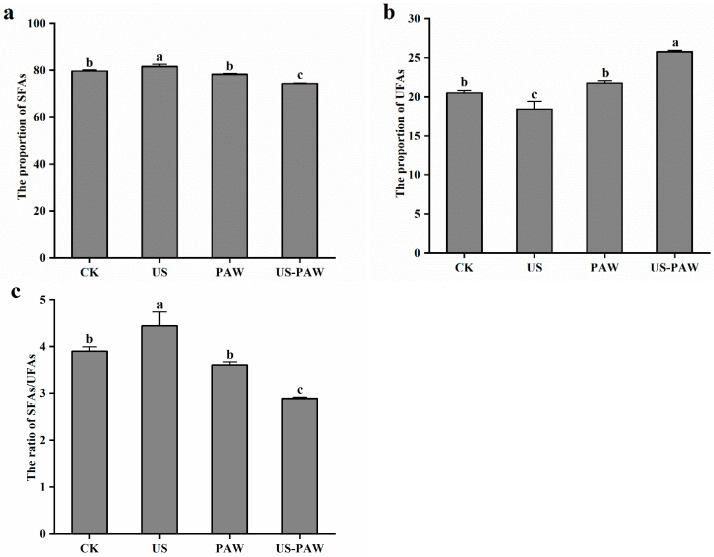
Effect of different treatments on the proportion of SFAs (**a**), UFAs (**b**), and the ratio of SFAs/UFAs (**c**) in *E. coli*. Values are shown as the means ± SD. Different small letters in each group indicate significant (*p* < 0.05) statistical differences. CK: control group. US: ultrasound. PAW: plasma-activated water. US-PAW: US combined with PAW. SFAs: saturated fatty acids. UFAs: unsaturated fatty acids.

Previous studies have shown that unsaturated branched-chain fatty acids increase cell membrane fluidity, while saturated long-chain fatty acids decrease cell membrane fluidity [43]. Thus, the ratio of SFAs/UFAs was used to express the membrane fluidity, and an increase in the ratio indicated a decrease in the fluidity of the cell membrane. The ratio of SFAs/UFAs was measured in the present study. As depicted in Figure 8, US treatment changed the proportion of membrane fatty acid composition, increasing the ratio of SFAs/UFAs. This suggests that the fluidity of *E. coli* cell membranes decreased after US treatment, resulting in a defensive state for the cells as they resisted environmental stressors. After US-PAW treatment, the ratio of SFAs/UFAs dropped to 2.88 ± 0.03%, representing a 20.00% reduction compared to PAW treatment alone. This showed that, after US-PAW treatment, the membrane fluidity of *E. coli* was promoted and the ability to resist external conditions was reduced, resulting in the exchange of substances inside and outside the cell enhanced, affecting cellular physiological metabolism.

#### 3.5.5. Membrane Protein Structure

The effect of US and PAW on the membrane protein structure of *E. coli* can be evaluated by endogenous fluorescence intensity. Amino acid residues, including tryptophan (Trp), tyrosine (Tyr), and phenylalanine (Phe), are the principal fluorophore in membrane proteins [44]. KI can quench the fluorescence of surface residues of membrane protein; however, it does not affect the fluorescence spectrum of internal residues of membrane protein. Thus, based on the effect of KI on the fluorescence of these amino acid residues, the positions of Trp, Tyr, and Phe can be qualitatively defined [45].

**Figure 9:**
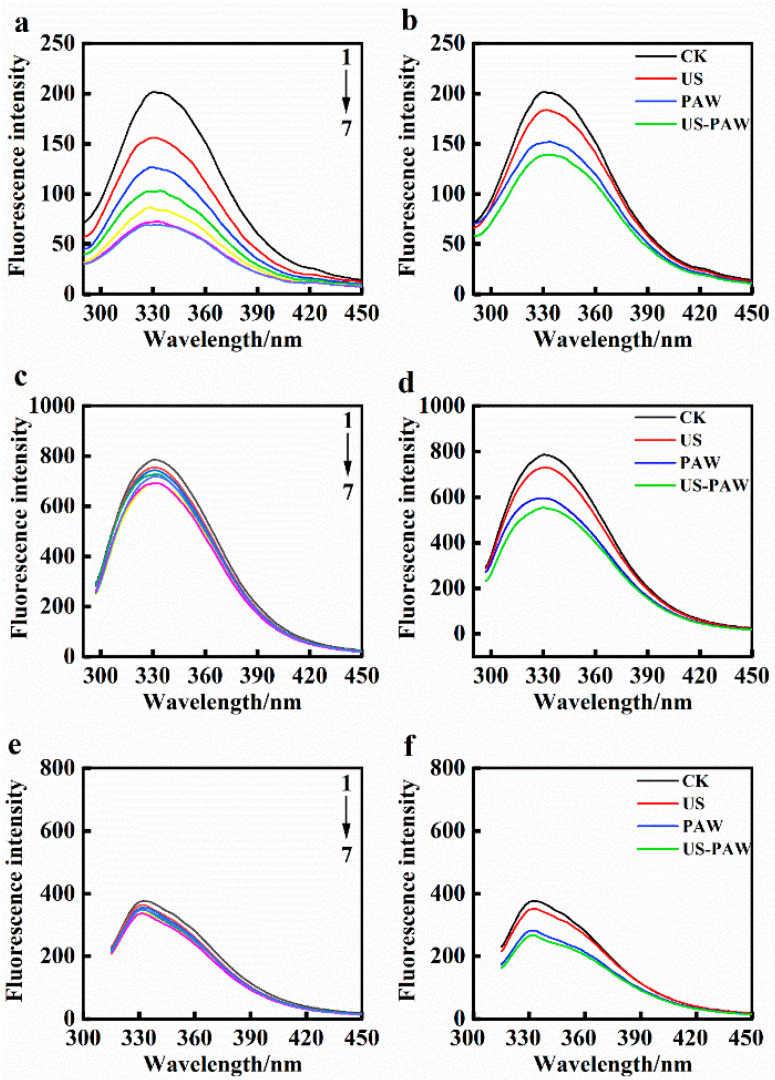
Effect of KI (**a**,**c**,**e**) and different treatments (**b**,**d**,**f**) on fluorescence intensity of amino acid residues Phe ((**a**,**b**), λex = 258 nm), Trp ((**c**,**d**), λex = 280 nm), and Tyr ((**e**,**f**), λex = 296 nm), respectively. Concentration of KI (from the top down): 0, 5, 10, 15, 20, 25, 30 mM. CK: control group. US: ultrasound. PAW: plasma-activated water. US-PAW: US combined with PAW.

The fluorescence emission spectra of Phe, Trp, and Tyr residues in *E. coli* membrane proteins were influenced by KI (Figure 9a,c,e). With the increase in KI concentration, the fluorescence intensity of Phe decreased significantly, indicating that Phe residues were mainly located externally. Trp and Tyr residues had no significant quenching effect, indicating that Trp and Tyr residues were mainly located in the membrane. The results were consistent with previous studies [26]. The effect of different treatments on the fluorescence spectra of amino acid residues Phe, Trp, and Tyr of *E. coli* is shown in Figure 9b,d,f. Compared to US and PAW single treatment, the maximum emission peak of amino acid residues in the US-PAW group was apparently reduced, indicating the membrane protein structure was modified. It also suggested that PAW combined with US treatment showed a mutually reinforcing effect. For Trp and Tyr residues, the fluorescence peak of the US-PAW group was lower than that of the maximum KI concentration, indicating that compared to KI, the US-PAW treatment could more significantly alter the cell membrane protein structure. At the same time, after US-PAW treatment, the fluorescence spectra displayed a slight blueshift, suggesting that the US-PAW treatment acted on cell membrane proteins, causing conformational changes in the proteins, changing the microenvironment of Phe, Trp, and Tyr. The stability of membrane protein conformation is essential for bacterial growth and viability [46]. This study was consistent with the results of US combined with sodium hypochlorite [47]; by combining US treatment, the fluorescence peak was reduced so that the structure of cell membrane proteins was altered, ultimately leading to chaos, decomposition, and death.

### 3.6. Effect of US-PAW Treatment on Oxidative Damage of E. coli

**Figure 10:**
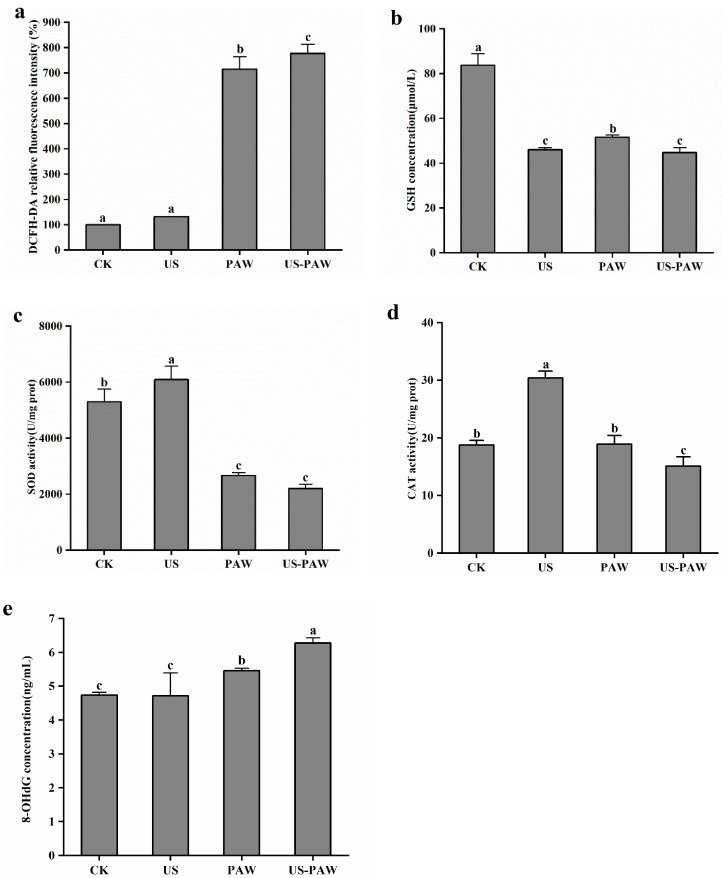
Effect of different treatments on the level of intracellular ROS (**a**), GSH concentration (**b**), SOD activity (**c**), CAT activity (**d**), and 8-OHdG concentration (**e**). Values are shown as the means ± SD. Different small letters in each group indicate significant (*p* < 0.05) statistical differences. CK: control group. US: ultrasound. PAW: plasma-activated water. US-PAW: US combined with PAW.

The content of ROS is an important signal of normal physiological function. As displayed in Figure 10, there was no significant (*p* > 0.05) increase in *E. coli* in the relative fluorescence intensity of the US group compared with the control. This was contrary to the results of Duan et al. [48]; it may be due to the lower ultrasonic frequency treated with *E. coli*, which failed to directly damage cells and induce intracellular ROS accumulation. However, the level of ROS increased significantly (*p* < 0.05) with PAW and US-PAW treatment. The relative fluorescence intensity in the US-PAW group was 8.70% higher than that in the PAW group, showing significant differences (*p* < 0.05). It indicated that the combined treatment caused a severe oxidative stress response in the cells. Compared with the control group, the cells after collaborative treatment showed a higher ROS level, which may be due to the rapid opening of cell channels by US, resulting in the oxidation reaction of a large number of active substances in PAW in the cell and the generation of a large number of ROS [49].

Additionally, due to the thin plasma membrane and cell wall of Gram-negative bacteria, the excessive generated ROS leads to the loss of permeability of the damaged cell membrane, and intracellular substances leak out from both, thereby causing irreversible damage and cell death [50]. This result was consistent with the increasing trend of cell membrane permeability.

The content of GSH, the activity of SOD, and the activity of CAT are reflected the level of intracellular antioxidants. As displayed in Figure 10b, compared with CK group, the GSH content in *E. coli* treated with three treatments was significantly (*p* < 0.05) reduced. The US-PAW treatments resulted in a reduction of intracellular GSH content to 44.71 ± 2.09 μM, which was significantly (*p* < 0.05) lower than that in the PAW group. SOD is an antioxidant metalloenzyme that is considered as the first line of defense against ROS, and its activity largely determines the resistance of bacteria to oxidative stress. SOD catalyzes the disproportionation of superoxide anion radicals to generate O_2_ and H_2_O_2_, which are central regulators of microbial ROS levels [51]. As shown in Figure 10c, intracellular antioxidant enzymes were activated and SOD activity significantly (*p* < 0.05) increased after US treatment. PAW and US-PAW treatments significantly (*p* < 0.05) decreased the SOD activity compared to CK. The SOD activity of *E. coli* was decreased by 58.40% with US-PAW treatment, compared with CK. As shown in Figure 10d, the CAT activity significantly (*p* < 0.05) increased when US processing was conducted, and the organism mobilized CAT enzyme for decomposing hydrogen peroxide in response to oxidative damage caused by free radicals, whereas the CAT activity declined significantly (*p* < 0.05) by 19.50% with US-PAW treatments. When cells are invaded by external factors, the intracellular antioxidant enzymes of *E. coli* are activated, thereby playing a defensive role in the cell. After PAW treatment, the SOD activity was enhanced, suggesting the cell was in a defensive state and achieved defense by increasing energy metabolism. The US-PAW treatment significantly reduced the activities of SOD and CAT, as well as GSH content, indicating that the active substances produced by collaborative treatment can lead to a decrease in intracellular antioxidant enzyme activity, resulting in damage to the antioxidant enzyme system.

As displayed in Figure 10e, there was no significant (*p* > 0.05) increase in *E. coli* in the 8-OHdG concentration of US group compared with the control. After PAW and US-PAW treatment, the 8-OHdG concentration was significantly (*p* < 0.05) increased. The concentration of 8-OHdG in the US-PAW treatment was 6.28 ± 0.16 ng/mL, which significantly (*p* < 0.05) increased by 33.05% and 15.02%, compared with the PAW and US group, respectively. The results showed that the combined treatment caused oxidative damage to DNA in *E. coli* cells. This was due to excessive ROS attacking the DNA, resulting in genetic information disorder [52], consistent with a previous report. Ki et al. reported that PAW treatment shows good bactericidal ability against *Aspergillus brasiliensis* and leads to the degradation of genomic DNA [53].

### 3.7. Correlation Analysis

To further clarify the relationship between bacterial log reduction, SI rate, A260, A280, relative fluorescence intensity of PI and DiBAC_4_(3), membrane fatty acid composition, amino acid residues, and ROS level, antioxidant oxidase activity, the 8-OHdG concentration, Spearman’s correlation analysis was conducted in the present study.

**Figure 11:**
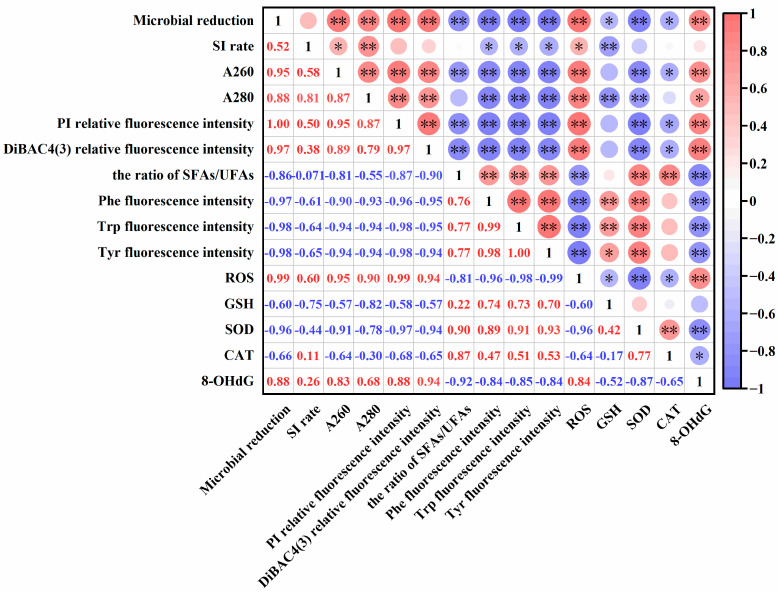
Correlation analysis of microbial log reduction with the cell membrane structure and oxidant damage. * *p* < 0.05, ** *p* < 0.01. SI: sublethal injury. A260 and A280: the leakage of nucleic acid and protein, respectively. SFAs: saturated fatty acids. UFAs: unsaturated fatty acids.

As shown in Figure 11, there was a positive correlation between bacterial log reduction and A260, A280, fluorescence intensity of PI, fluorescence intensity of DiBAC_4_(3), ROS content, and 8-OHdG concentration. Among them, the correlation coefficient between bacterial log reduction and cell membrane permeability was relatively high, reaching 0.998. Cell membrane permeability had a significant positive correlation with the leakage of protein and nucleic acid, as well as cell membrane potential. ROS content showed a significant negative correlation with the ratio of SFAs/UFAs, amino acid residues fluorescence intensity, GSH level, SOD activity, and CAT activity, and a significant positive correlation with the 8-OHdG concentration. In addition, the SI rate was positively correlated with A260, A280, and ROS content, and negatively correlated with GSH content and amino acid residue fluorescence intensity. These results indicated cell membrane structure and cellular oxidative state were responsible for the antibacterial effect.

In the present study, the effect of US combined PAW treatment on the inhibitory efficiency of *E. coli* was investigated, focusing on reducing bacterial count and SI rate. The results showed that, after plasma activation for 10 min and combination with US treatment for 10 min, the US-PAW treatment significantly increased cell membrane structure destruction and induced oxidant damage, ultimately causing irreversible damage and cell death to *E. coli*, thereby reducing the SI rate of the bacteria while increasing log reduction. In practice, reducing SI rates plays a critical role in effectively inactivating damaged cells, thereby preventing their resuscitation and ultimately ensuring food safety [11]. This provides important assistance in controlling the microbial by US-PAW treatment in food processing applications, such as cleaning fruits, vegetables, aquatic products.

Nevertheless, there were some limitations in the present study. This study only explored the effects of US-PAW combined treatment on Gram-negative bacteria. Further research is needed on the bactericidal mechanism of US-PAW treatment on Gram-positive bacteria and the inhibitory effect on SI. Also, the current study only examined with pure bacterial suspensions, which may not fully represent real food environments. Further studies should validate the technique across the application of US-PAW treatment in different food matrices, such as fruit, vegetable, poultry, and aquatic products, including its impact on food quality and changes in microorganisms during the storage period.

## 4. Conclusions

This study explored the lethal and sublethal effect of US combined PAW treatment on *E. coli*. After plasma activation 10 min and combination with US treatment for 10 min, US-PAW treatment significantly increased cell membrane structure destruction and meditated oxidant damage, causing irreversible damage, and reducing microbial count while controlling the SI of the microorganisms. In this hurdle technology, PAW played the principal role in microbial log reduction, and US treatment acted in reducing the SI rate. This study finding provides better guidance for increasing the practical application of US-PAW processing. However, the effect of US-PAW treatment on the quantity of microorganisms in different food matrices and the storage period on their quality remains to be observed.

## Figures and Tables

**Table 1:** Microbial log reduction in *E. coli* under different treatments.

Treatments	Microbial Log Reduction (log CFU/mL)			
	3 min	5 min	7 min	10 min
US	0.01 ± 0.01 Cd	0.03 ± 0.01 Ce	0.13 ± 0.03 Bf	0.19 ± 0.04 Ag
PAW5	0.26 ± 0.04 Cc	0.30 ± 0.01 Cde	0.48 ± 0.07 Be	0.86 ± 0.13 Af
PAW10	0.75 ± 0.12 Db	1.66 ± 0.06 Cc	2.49 ± 0.12 Bc	3.59 ± 0.08 Ad
PAW15	1.03 ± 0.15 Da	1.74 ± 0.02 Cbc	3.32 ± 0.22 Bb	4.83 ± 0.07 Ac
US-PAW5	0.31 ± 0.02 Cc	0.49 ± 0.12 Cd	0.87 ± 0.07 Bd	2.12 ± 0.19 Ae
US-PAW10	1.12 ± 0.15 Da	1.99 ± 0.28 Cb	2.71 ± 0.19 Bc	4.89 ± 0.07 Ab
US-PAW15	1.25 ± 0.14 Da	2.43 ± 0.17 Ca	3.75 ± 0.06 Ba	5.24 ± 0.40 Aa

Note: Values followed by different capital letters in the same row indicate statistically significant differences (*p* < 0.05). Values followed by different lowercase letters in the same column indicate statistically significant differences (*p* < 0.05). US: ultrasound. PAW: plasma-activated water. US-PAW: US combined with PAW. The numbers 5, 10, and 15 represent plasma activation at 5 min, 10 min, and 15 min.

**Table 2:** SI rate of *E. coli* under different treatment.

Treatments	SI Rate (%)			
	3 min	5 min	7 min	10 min
US	23.80 ± 2.10 Bd	22.28 ± 1.45 BCc	51.18 ± 5.21 Aa	16.13 ± 3.25 Cc
PAW5	42.15 ± 2.11 Ab	38.15 ± 1.40 ABb	35.64 ± 4.01 BCb	31.01 ± 1.59 Cb
PAW10	45.21 ± 3.12 Ab	37.31 ± 2.24 Bb	34.25 ± 1.45 Bb	28.14 ± 1.45 Cb
PAW15	77.09 ± 2.95 Aa	65.51 ± 5.20 Ba	54.52 ± 5.01 Ca	40.48 ± 3.25 Da
US-PAW5	41.14 ± 4.21 Ab	41.41 ± 2.64 Ab	32.15 ± 1.48 Bb	31.02 ± 4.15 Bb
US-PAW10	45.52 ± 3.41 Ab	35.91 ± 1.43 Bb	35.04 ± 6.05 Bd	13.30 ± 2.15 Cc
US-PAW15	32.09 ± 2.63 Cc	62.21 ± 5.41 Aa	50.56 ± 3.41 Ba	29.30 ± 4.17 Cb

Note: Values followed by different capital letters in the same row indicate statistically significant differences (*p* < 0.05). Values followed by different lowercase letters in the same column indicate statistically significant differences (*p* < 0.05). SI: sublethal injury. US: ultrasound. PAW: plasma-activated water. US-PAW: US combined with PAW. The numbers 5, 10, and 15 represent plasma activation at 5 min, 10 min, and 15 min.

**Table 3:** Kinetics parameters calculated by two mathematics models for the microbial reduction in *E. coli* treated by US, PAW, and US-PAW.

Treatments	Linear Model		Weibull Model		
	D	R^2^	σ	ρ	R^2^
US	114.9186	0.4726	19.3897	2.3447	0.8921
PAW5	15.8633	0.7481	12.1841	1.3172	0.8127
PAW10	8.5232	0.7950	7.0133	1.4392	0.9272
PAW15	2.8332	0.9861	3.2383	1.1375	0.9965
US-PAW5	2.6605	0.9988	2.5542	0.9692	0.9992
US-PAW10	2.7765	0.8856	3.2828	1.3296	0.9827
US-PAW15	1.5255	0.8826	2.1950	1.3236	0.9195

Note: US: ultrasound. PAW: plasma-activated water. US-PAW: US combined with PAW. The numbers 5, 10, and 15 represent plasma activation at 5 min, 10 min, and 15 min.

**Table 4:** The changes in membrane fatty acid composition of *E. coli* after different treatments.

Fatty Acids (%)	CK	US	PAW	US-PAW
C4:0	18.83 ± 1.95 a	13.12 ± 1.36 ab	7.75 ± 5.15 b	8.04 ± 0.01 b
C12:0	0.53 ± 0.09 c	1.17 ± 0.01 c	2.87 ± 0.64 b	4.17 ± 0.45 a
C14:0	3.15 ± 0.84 a	3.26 ± 0.51 a	3.27 ± 0.14 a	3.21 ± 0.39 a
C15:0	3.42 ± 0.96 a	3.54 ± 0.59 a	4.17 ± 0.29 a	4.47 ± 0.56 a
C16:0	46.71 ± 0.37 b	53.09 ± 1.76 a	49.46 ± 2.86 b	38.26 ± 0.02 c
C17:0	1.91 ± 0.50 a	1.95 ± 0.34 a	2.20 ± 0.17 a	2.14 ± 0.23 a
C18:0	3.19 ± 0.09 b	3.14 ± 0.41 b	4.71 ± 0.02 a	5.31 ± 0.43 a
C16:1	13.56 ± 0.65 a	9.03 ± 0.73 b	12.69 ± 0.79 a	14.07 ± 1.13 a
C18:1	1.02 ± 0.31 b	1.14 ± 0.05 ab	0.85 ± 0.27 b	1.69 ± 0.31 a
C18:2	3.56 ± 0.44 b	4.21 ± 0.36 ab	3.74 ± 0.22 ab	4.46 ± 0.21 a

Note: Values followed by different lowercase letters in the same row indicate statistically significant differences (*p* < 0.05). US: ultrasound. PAW: plasma-activated water. US-PAW: US combined with PAW.

## Data Availability

The original contributions presented in the study are included in the article, further inquiries can be directed to the corresponding author.

## Abbreviations

The following abbreviations are used in this manuscript:

PAW	plasma-activated water
US-PAW	ultrasound Combined with plasma-activated water
US	ultrasound
*E. coli*	*Escherichia coli*
SI	sublethal injury
